# Evaluation of Metabolic Enzymes in Response to Excel Mera 71, a Glyphosate-Based Herbicide, and Recovery Pattern in Freshwater Teleostean Fishes

**DOI:** 10.1155/2014/425159

**Published:** 2014-06-12

**Authors:** Palas Samanta, Sandipan Pal, Aloke Kumar Mukherjee, Apurba Ratan Ghosh

**Affiliations:** ^1^Ecotoxicology Laboratory, Department of Environmental Science, The University of Burdwan, Golapbag, Burdwan, West Bengal 713104, India; ^2^Department of Environmental Science, Aghorekamini Prakashchandra Mahavidyalaya, Subhasnagar, Bengai, Hooghly, West Bengal 712611, India; ^3^P.G. Department of Conservation Biology, Durgapur Government College, Durgapur, West Bengal 713214, India

## Abstract

Metabolic enzymes such as alanine aminotransferase (ALT), aspartate aminotransferase (AST), and alkaline phosphatase (ALP) were evaluated in Indian teleostean fishes, namely, *Anabas testudineus* (Bloch) and *Heteropneustes fossilis* (Bloch), for an exposure to 30 days of Excel Mera 71 (17.2 mg/L), a glyphosate formulation, and subsequent depuration under Liv.52, a plant extract at a dose of 187.5 mg/d/250 L for the same period in the same tissues under laboratory condition. ALT activity was significantly increased (*P* < 0.05) in all the tissues and raised up to 229.19% in liver of *A. testudineus* (229.19%) and 128.61% in liver of *H. fossilis*. AST also increased significantly (*P* < 0.05) and was maximum in liver of *H. fossilis* (526.19%) and minimum in gill of *A. testudineus* (124.38%). ALP activity was also raised highly in intestine of *H. fossilis* (490.61%) but was less in kidney of *H. fossilis* (149.48%). The results indicated that Excel Mera 71 caused alterations in the metabolic enzymatic activities in fish tissues and AST showed the highest alteration in both the fishes, while lowest in ALP and ALT in *A. testudineus* and *H. fossilis*, respectively. During depuration under Liv.52, all the enzyme activities came down towards the control condition which indicated the compensatory response by the fish against this herbicidal stress and it was in the following order: AST > ALT > ALP, in *A. testudineus*, while *H. fossilis* showed the following trend: ALT > AST > ALP. Therefore, these parameters could be used as indicators of herbicidal pollution in aquatic organisms and were recommended for environmental monitoring for investigating the mechanism involved in the recovery pattern.

## 1. Introduction

Pesticidal contamination in the aquatic ecosystem has gained much attention all around the world in the last few decades as they are posing serious threat to the aquatic organisms especially nontarget organisms, namely, fin fishes and shell fishes, which are exposed to large amount of pesticides continuously as a consequence of rapid industrialization, agricultural practices, and other anthropogenic activities. Herbicide induces damage at histological, physiological, metabolic, and biochemical levels of organisms due to its higher concentrations or its persistence in the aquatic medium, ultimately leading to changes in function of the cells, tissues, and behaviour of the organism [1, 2]. Fishes are most vulnerable to these toxicants because it is believed that, regardless of the sources of pollution, they will eventually end up in the aquatic environment and they are usually the final consumer in that medium.

Excel Mera 71, a glyphosate-based herbicide formulation, is widely used as nonselective and postemergent herbicide in our regions for controlling weeds in agriculture, forestry, urban areas, and even in aquatic bodies. It is a new 71% water-dispersible granule (WDG) herbicide formulation of ammonium salt of glyphosate and is toxic to aquatic organisms [3]. Glyphosate formulations do not bioaccumulate in terrestrial or aquatic animals and have the potentiality for rapid dissipation in waters [3]. It is used throughout the world due to its high efficiency, cost effectiveness, and biodegradability in the environment by soil microflora which converts it into aminomethylphosphonic acid (AMPA) and CO_2_ [3, 4].

Effects of Roundup herbicide, a glyphosate-based formulation on metabolic and enzymatic activities in freshwater fishes, have been observed by researchers worldwide [5–7]. Transamination, regulated by aminotransferases (alanine aminotransferases (ALT) and aspartate aminotransferase (AST)), is one of the principal pathways for the synthesis and deamination of amino acids, thereby, allowing interconversion between carbohydrate and protein metabolism during stress imposed conditions to meet the high energy demand of the organisms [8], while transphosphorylation is controlled by alkaline phosphatase, a polyfunctional enzyme that acts at alkaline pH and plays a vital role in the mineralization of the skeleton of aquatic organisms and membrane transport activities [9]. Therefore, changes in the biochemical parameters by environmental stressors indicate alterations in metabolism and biochemical processes of the organisms and measurement of these parameters can be used as the diagnostic tools in fish toxicology to measure the fish health status and to identify the extent of damage at target organs exposed to herbicides [9].

The Liv.52 is the approved ayurvedic medicine by drug regulatory authority, Department of AYUSH, Ministry of Health and Family Welfare, Government of India. Each tablet of Liv.52 contains extracts of the following medicinal plants in definite proportions in powder form: Himsra (*Capparis spinosa*) 65 mg, Kasani (*Cichorium intybus*) 65 mg, Mandur bhasma 33 mg, Kakamachi (*Solanum nigrum*) 32 mg, Arjuna (*Terminalia arjuna*) 32 mg, Kasamarda (*Cassia occidentalis*) 16 mg, Biranjasipha (*Achillea millefolium*) 16 mg, and Jhavuka (*Tamarix gallica*) 16 mg. It is rich in phenolic compounds particularly polyphenols and is used since a long time to combat liver disorders and plays a pivotal role in detoxification of xenobiotics from liver [10–12] both in human and animal models. Few studies, however, have been reported to address the hepatoprotective effects of Liv.52 in rat to establish the correlation between the hepatoprotective potency and biological activity [10–12].

Most of the studies are confined to investigate the biochemical and physiological changes of these xenobiotics, namely, herbicides, but very little attention was given to draw a comparative analysis in between the two conditions of herbicide intoxication and depuration under Liv.52 in two fish species, namely,* Anabas testudineus* and* Heteropneustes fossilis*, in regard to metabolic enzymes. Finally, these enzymes may be used as bioindicators in herbicidal intoxication. Therefore, the objective of the present study is to analyse the biochemical alterations in different tissues of* A. testudineus* and* H. fossilis* exposed to glyphosate and subsequent recovery pattern in herbicide-free water when supplemented with Liv.52 on comparative basis.

## 2. Materials and Methods

### 2.1. Experimental Design

Freshwater teleosts,* Anabas testudineus *(Bloch) and* Heteropneustes fossilis* (Bloch), of both sexes with an average weight of 16.09 ± 1.42 g and 25.33 ± 1.50 g, respectively, and total length of 10.09 ± 0.25 cm and 17.03 ± 0.35 cm, respectively, were procured from the local market and were acclimatized to congenial laboratory conditions for 15 days separately in aquaria of 250 L capacity. After 15 days, fishes were divided into two exposure sets (control and Excel Mera 71-treated) and maintained in eight aquaria, containing 10 fishes in each aquarium at the Ecotoxicology Laboratory, Department of Environmental Science, The University of Burdwan: three for* A. testudineus*, three for* H. fossilis*, and two control sets (*A. testudineus* and* H. fossilis*). The fishes were exposed to a sublethal dose of Excel Mera 71, that is, 17.20 mg/L (20% of the 96 h LC_50_ value in* Oncorhynchus mykiss*, that is, 86 mg/L) [13], in 250 L aquaria for a period of 30 days. Doses were applied on every alternate day basis. During the period of experiment both the treated and control sets of fishes were fed with minced goat liver regularly. Cleaning, dewatering, renewing with freshwater, and feeding were maintained regularly on every alternate day basis. The aerator bubble was also provided in all aquaria to avoid any oxygen depletion. During the experimentation, the same environmental conditions are maintained in all aquaria throughout the exposure period. Experiments were conducted at natural photoperiod. After an exposure period of 30 days, on 31st day, five fishes from each treated aquarium were collected and sacrificed and desired tissues (gill, intestine, liver, kidney, heart, and muscles) were taken, while, in case of control, ten fishes were sacrificed and washed in 0.75% saline solution, blotted with tissue paper and kept in teflon tubes, and finally stored at −20°C for posterior analysis.

The remaining five fishes from each treated set were transferred to the separate aquaria under Liv.52 (mixture of caper bush and chicory) at a dose of 187.5 mg/d/250 L for another 30 days as recovery period. Maintenance of each aquarium and collection of tissue samples were the same as before the 31st day.

### 2.2. Tissue Preparation

The desired tissues were taken out from the freezer for analysis and soaked with tissue paper. Then tissues were weighed maximum up to 0.1 g and homogenized in 2 mL of 0.5 M (pH 7.4) Tris-HCl buffer by using mortar and pestle and finally centrifuged at 8,000 rpm for 25 minutes at 0°C and the supernatant was taken in teflon tubes for the analysis of alanine aminotransferase, aspartate aminotransferase, and alkaline phosphatase in both conditions and recorded in the Tables 1, 2, and 3, respectively.

### 2.3. Biochemical Parameters

Alkaline phosphatase (ALP) activity was measured according to the method of Bergmeyer et al. [14] by using MERCK kit (Merck cat. number 1730PDLFT.0045). Briefly, 400 *μ*L of R1 reagent was taken in the test tube and then 100 *μ*L of R2 reagent was added to this solution. These two solutions were mixed up and kept for incubation at 37°C for 60 seconds. After that, 10 *μ*L of sample was added and immediately reading was taken from the autoanalyzer for four minutes. Enzyme activity was expressed as IU/L. Activity of aspartate aminotransferase (AST) was determined following the procedure of Bergmeyer et al. [14] by using Erba kit (Erba cat. number FBCEM0045). In brief, 500 *μ*L of R1 reagent was taken in test tube and kept for incubation at 37°C. Then 50 *μ*L of sample was added and immediately reading was taken from the autoanalyzer for three minutes. Enzyme activity was expressed as IU/L. Tissue alanine aminotransferase (ALT) was measured according to the method described by Bergmeyer et al. [14] using Erba kit (Erba cat. number FBCEM0047) and the reading was taken from the autoanalyzer. All the assays were run in triplicate to avoid the error as much as possible.

### 2.4. Data Analysis

The data were statistically analyzed using SPSS package (version 16). One-way analysis of variance (ANOVA) was applied to compare the mean values of different parameters among different tissues, concentrations, and fishes. Mean values were compared by Tukey's test. The values of all the biochemical parameters were expressed as mean ± SE (*n* = 15 for both the treatments and *n* = 10 for control). A *P* < 0.05 was considered statistically significant.

### 2.5. Ethical Statement

The experiment was carried out in accordance with the guidelines of the University of Burdwan and approved by the Ethical Committee of this university.

## 3. Results and Discussion

The present study is mainly concerned with comparative evaluation of enzymatic activities after glyphosate intoxication and subsequent recovery under Liv.52 in Excel Mera 71-free water in different tissues, namely, liver, muscle, gill, heart, kidney, and intestine, and so forth, of two Indian air-breathing teleosts,* A. testudineus *and* H. fossilis*.

Alanine aminotransferase (ALT) plays a vital role in synthesis and deamination of amino acids during stress imposed conditions for meeting the high energy demand of the organism [8]. In the present study, ALT activity after exposition of Excel Mera 71 in the laboratory condition was significantly increased (*P* < 0.05) in all the tissues of the test fishes when compared to control value (Table 1). In case of liver, ALT activity in* A. testudineus* was 229.19% and 128.61% in* H. fossilis*. Heart tissue also showed similar results as in liver such as 226.05% and 194.12% in* A. testudineus* and* H. fossilis*, respectively. In case of muscle and gill tissue, ALT activity was 181.47% and 218.75%, respectively, in* H. fossilis* but, in* A. testudineus*, it was 142.96% and 172.46%, respectively. The results clearly depicted that ALT activity was raised highly in liver of* A. testudineus*, that is, 229.19%, and was less in liver of* H. fossilis* (128.61%) but this increased activity was not species specific; it is not even tissue specific. The increased activity of ALT in different tissues of the test fishes in the present study indicated tissue damage which may be due to disturbance in normal physiological and biochemical processes such as Krebs' cycle, TCA cycle, and subsequent leakage of this enzyme from the liver cytosol through membrane into the blood stream. The present result was in agreement with the results of Jee et al. [15] who observed increased activity of serum ALT in Korean rockfish,* Sebastes schlegeli*, exposed to cypermethrin. Several authors also observed increased activity of ALT as a consequence of pesticide exposure in teleostean fishes [16–18]. Enhanced activity of ALT provided the oxaloacetic acid and pyruvate to meet the increased energy demand during carbofuran imposed stress condition in the fish* Clarias batrachus* [18]. However, during the recovery period, ALT activity was reduced in comparison to the treated condition and approaching towards the control value in all the investigated tissues of both the test fish species after 30 days, that is, in the depuration phase under herbicide-free water supplemented with Liv.52. In case of* A. testudineus*, recovery pattern was in the following order: gill (67.69%) > heart (61.94%) > muscle (57.06%) > liver (52.04%), while in* H. fossilis* the pattern was as follows: muscle (72.06%) > gill (66.00%) > heart (57.57%) > liver (54.09%). Decreased ALT activity in all the tissues of test fishes in the recovery period could represent an induction of adaptive repairing mechanism by Liv.52 against the herbicide toxicity and could be considered as an effective detoxifying agent.

Aspartate aminotransferase (AST), although a liver specific enzyme, is found in high amounts in skeletal muscle cells and promotes gluconeogenesis from amino acids in association with ALT [19]. AST activity in liver, muscle, gill, and heart of the test fishes was measured after Excel Mera 71 exposure and at recovery phase with Liv.52. AST activity was increased significantly (*P* < 0.05) as compared to control value during Excel Mera 71 treatment but decreased during recovery phase (Table 2). AST activity after Excel Mera 71 exposition showed usual trend of enhancement as compared to control value. In the present study, liver tissue showed the highest elevation of AST activity in* H. fossilis* (526.19%) as compared to* A. testudineus* (367.49%), while, in heart, the highest activity was observed in* A. testudineus* (153.25%). In case of muscle and gill tissues, increased AST activity was 294.24% and 137.87%, respectively, in* H. fossilis* but AST activity was less in* A. testudineus*, that is, 234.05% and 124.38%, respectively. Considering all the tissues, here, the highest AST activity was observed in the liver of* H. fossilis* (526.19%) and the lowest in the gill of* A. testudineus* (124.38%) under Excel Mera 71 exposure. The study also showed that elevation pattern of enzyme activity was different for different fish tissues but it revealed that* H. fossilis *was more sensitive to the changes than* A. testudineus*. Enhanced AST activity in the investigated fish tissues under Excel Mera 71 toxication in the present study was an indication of damage either in tissues or organs leading to release of the enzyme into the serum or blood circulation and the presence of these metabolites acts as intermediate to the Krebs' cycle. Similar results of increased AST activity were also reported by different authors in different fish tissues exposed to pesticides [20, 21]. During recovery period, AST level came down and approached towards the normal value in all the tissues of both fishes during 30 days of depuration. The recovery pattern in* A. testudineus* was in the following order: gill (87.14%) > heart (72.72%) > muscle (61.36%) > liver (39.99%), while, in* H. fossilis*, it was as follows: gill (67.85%) > heart (61.66%) > muscle (38.72%) > liver (29.95%). Reduced activity of AST during the recovery period indicated counter response mechanism by the fishes to protect the permeability and integrity of membrane structure against herbicidal toxicity and to develop a compensatory response in the physiological system during depuration under Liv.52 which has the capability to protect the tissue functions against herbicidal toxicosis.

Alkaline phosphatase is considered as biomarker due to its adaptive response to the cytotoxic and genotoxic effects of xenobiotics [22] and plays a pivotal role in the transport of metabolites across the membranes. Alkaline phosphatase activity, likewise ALT and AST in liver, intestine, and kidney of both the fish species, was significantly increased (*P* < 0.05) after Excel Mera 71 exposure but decreased in recovery phase (Table 3). In liver, the highest increased ALP activity was recorded in* H. fossilis* (380.97%) followed by* A. testudineus* (219.66%) and similar pattern was also observed in intestine, while, in kidney, the highest increased activity of ALP was observed in* A. testudineus* (370.42%) and the lowest in* H. fossilis* (149.48%). Considering all the tissues, the highest ALP activity was observed in intestine of* H. fossilis *(490.61%) and the lowest in kidney of* H. fossilis *(149.48%). Alkaline phosphatase activity during Excel Mera 71 exposure in the present study was enhanced in all the fish tissues which may be due to cellular damage in tissue system which may indicate higher metabolic activities to meet the high energy demand to cope with herbicidal stress. The results were in agreement with the findings of Al-Attar [23] and Samanta et al. [24] but deferred with Das and Mukharjee [25] who observed declined ALP activity in brain of* Labeo rohita *exposed to cypermethrin. During the recovery period, ALP level was also reduced in similar pattern as in ALT and AST and came down towards the control value. The recovery pattern in case of* A. testudineus* was in the following order: liver (70.82%) > intestine (57.43%) > kidney (36.48%), while, in* H. fossilis*, it was in the following order: kidney (77.85%) > liver (56.05%) > intestine (38.06%). Like transaminases, ALP activity was also reduced during recovery phase as a compensatory response to overcome the stress. Reduced activity of ALP during recovery period may be due to lower synthesis rate of glycogen and less energy demand as fishes are in herbicide-free condition. The results were also supported by Shaffi [26]. The results clearly revealed that the alteration of enzyme activity during Excel Mera 71 intoxication in case of* A. testudineus* was in the following order: AST > ALT > ALP, while the pattern of alteration in* H. fossilis* was as follows: AST > ALP > ALT with respect to concerned tissues. Again, the degree of recovery responses of enzyme activities was different in different fish species during depuration period; in* A. testudineus*, it was in the following order: AST > ALT > ALP, while, in* H. fossilis*, it was as follows: ALT > AST > ALP. Therefore, alterations in the biochemical enzyme activity in these tissues may be considered as sensitive biomarkers in ecotoxicological studies as an early prediction of herbicidal toxicosis in aquatic organisms and ultimately may be used as a tool of ecomonitoring.

## 4. Conclusion

The present investigation demonstrates the alterations in biochemical enzyme activities of ALT, AST, and ALP in the fish tissues under the commercial herbicide formulation, Excel Mera 71, containing glyphosate which finally affected the fish health. The most severe alteration in enzyme activity was observed in liver and may be due to its prime role in detoxification of the compound. Although, after recovery, fishes showed positive trends of recovery and healing responses towards the normal enzyme status indicating adaptive response against the herbicidal toxicity. Therefore, Liv.52 plays a vital role in smearing the Excel Mera 71 toxicosis in the concerned tissues of the test fish species that significantly replenished the ALT, AST, and ALP levels and acts as a detoxifying agent. Therefore, these biochemical parameters can be considered as indicators for herbicidal toxicosis, although further studies are required for investigating the mechanism involved in this recovery pattern.

## Figures and Tables

**Table 1:** Analysis of activity of alanine aminotransferase (unit/mg protein/min) in various test fishes under control, treated, and recovery conditions.

Tissues	Concentration	Type of Fishes	
	(mg/L)	*A. testudineus *	*H. fossilis *
Liver	0	83.60 ± 3.60^a1A^	42.12 ± 1.81^b1A^
	17.2	191.60 ± 11.70^a2A^	54.17 ± 1.29^b1A^
	Recovery	99.70 ± 9.20^a3A^	29.30 ± 3.73^b1A^

Muscle	0	22.95 ± 2.05^a1B^	10.63 ± 0.85^a1B^
	17.2	32.81 ± 2.59^a1B^	19.29 ± 1.84^a1B^
	Recovery	18.72 ± 3.13^a1B^	13.90 ± 1.33^a1B^

Gill	0	6.56 ± 0.46^a1C^	2.69 ± 0.22^a1C^
	17.2	11.32 ± 0.65^a1C^	5.88 ± 0.47^a1C^
	Recovery	7.66 ± 0.81^a1C^	3.881 ± 0.20^a1C^

Heart	0	14.24 ± 2.08^a1D^	9.87 ± 1.22^a1D^
	17.2	32.19 ± 1.06^a1D^	19.16 ± 2.59^a1D^
	Recovery	19.94 ± 1.51^a1D^	11.03 ± 1.59^a1D^

Data are presented as mean ± SE. Values with different lowercase superscripts (alphabet) differ significantly (*P* < 0.05) between fishes within tissue and concentration. Values with different numeric superscripts differ significantly (*P* < 0.05) between concentrations within tissue and fishes. Values with different uppercase superscripts (alphabet) differ significantly (*P* < 0.05) between tissues within fishes and concentration.

**Table 2:** Analysis of activity of aspartate aminotransferase (unit/mg protein/min) in various test fishes under control, treated, and recovery conditions.

Tissues	Concentration	Type of Fishes	
	(mg/L)	*A. testudineus *	*H. fossilis *
Liver	0	96.90 ± 2.00^a1A^	102.70 ± 3.40^a1A^
	17.2	356.10 ± 19.80^a2A^	540.40 ± 22.90^b2A^
	Recovery	142.40 ± 12.20^a3A^	129.40 ± 13.30^b3A^

Muscle	0	111.90 ± 2.60^a1B^	107.60 ± 2.80^a1B^
	17.2	261.90 ± 21.60^a2B^	316.60 ± 11.50^a2B^
	Recovery	160.70 ± 15.60^a3B^	122.60 ± 22.80^b3B^

Gill	0	113.84 ± 4.68^a1C^	128.80 ± 3.92^a1C^
	17.2	141.59 ± 1.82^a1C^	177.58 ± 9.91^a1C^
	Recovery	123.38 ± 3.03^a1C^	120.49 ± 5.30^a1C^

Heart	0	74.69 ± 4.03^a1D^	36.40 ± 2.02^a1D^
	17.2	114.46 ± 5.21^a1D^	47.16 ± 4.99^b1D^
	Recovery	83.24 ± 5.36^a1D^	29.08 ± 1.49^a1D^

Data are presented as mean ± SE. Values with different lowercase superscripts (alphabet) differ significantly (*P* < 0.05) between fishes within tissue and concentration. Values with different numeric superscripts differ significantly (*P* < 0.05) between concentrations within tissue and fishes. Values with different uppercase superscripts (alphabet) differ significantly (*P* < 0.05) between tissues within fishes and concentration.

**Table 3:** Analysis of activity of alkaline phosphatase (unit/mg protein/min) in various test fishes under control, treated, and recovery conditions.

Tissues	Concentration	Type of Fishes	
	(mg/L)	*A. testudineus *	*H. fossilis *
Liver	0	2.49 ± 0.36^a1A^	14.87 ± 1.20^a1A^
	17.2	5.46 ± 0.85^a1A^	56.65 ± 2.62^b2A^
	Recovery	3.87 ± 0.40^a1A^	31.75 ± 6.29^a3A^

Intestine	0	91.60 ± 3.40^a1B^	36.20 ± 2.20^a1B^
	17.2	148.80 ± 13.20^a2B^	177.60 ± 48.10^b1B^
	Recovery	85.45 ± 6.75^a1B^	67.60 ± 2.80^a1B^

Kidney	0	72.00 ± 14.70^a1C^	50.53 ± 3.19^a1C^
	17.2	266.70 ± 18.80^a1C^	75.53 ± 6.74^a1C^
	Recovery	97.30 ± 4.10^a1C^	58.80 ± 17.40^a1C^

Data are presented as mean ± SE. Values with different lowercase superscripts (alphabet) differ significantly (*P* < 0.05) between fishes within tissue and concentration. Values with different numeric superscripts differ significantly (*P* < 0.05) between concentrations within tissue and fishes. Values with different uppercase superscripts (alphabet) differ significantly (*P* < 0.05) between tissues within fishes and concentration.
